# Digestive microbiota is different in pigs receiving antimicrobials or a feed additive during the nursery period

**DOI:** 10.1371/journal.pone.0197353

**Published:** 2018-05-25

**Authors:** Cassandra Soler, Tim Goossens, Alvaro Bermejo, Lourdes Migura-García, Anna Cusco, Olga Francino, Lorenzo Fraile

**Affiliations:** 1 Departament de Ciencia Animal, ETSEA, Universitat de Lleida, Lleida, Spain; 2 Nutriad International N.V., Dendermonde, Belgium; 3 IRTA, Centre de Recerca en Sanitat Animal (CReSA, IRTA-UAB), Campus de la Universitat Autònoma de Barcelona, Bellaterra, Spain; 4 Servicio Veterinario de Genética Molecular, Facultad de Veterinaria, Campus de la Universitat Autònoma de Barcelona, Bellaterra, Spain; Wageningen Universiteit, NETHERLANDS

## Abstract

Antimicrobials have been used in a prophylactic way to decrease the incidence of digestive disorders during the piglet post-weaning period. Nowadays, it is urgent to reduce their consumption in livestock to address the problem of antimicrobial resistance. In this study, the effect of a product on piglet microbiota has been investigated as an alternative to antimicrobials. Three groups of ten post-weaning pigs were sampled at 0, 15 and 30 days one week post-weaning; the control, antibiotic and feed additive group received a standard post-weaning diet without antibiotics or additives, the same diet as the control group but with amoxicillin and colistin sulphate and the same diet as the control group but with a feed additive (Sanacore-EN, Nutriad International N.V.), respectively. The total DNA extracted from faeces was used to amplify the 16S RNA gene for massive sequencing under manufacturer’s conditions. Sequencing data was quality filtered and analyzed using QIIME software and suitable statistical methods. In general terms, age modifies significantly the microbiota of the piglets. Thus, the oldest the animal, the highest bacterial diversity observed for the control and the feed additive groups. However, this diversity was very similar in the antibiotic group throughout the trial. Interestingly, a clear increase in abundance of *Bacillus* and *Lactobacillus spp* was detected within the feed additive group versus the antibiotic and control groups. In conclusion, the feed additive group had a positive effect in the endogenous microbiota of post-weaning pigs increasing both, the diversity of bacterial families and the abundance of lactic acid bacteria during the post-weaning period.

## Introduction

Microbiota plays probably a significant role in host health and metabolism and the swine digestive tract provides the appropriate habitat for a huge number of microbial species. Historically, the identification of porcine microbiota has been carried out using culture-dependent techniques of which the ability to understand the microorganism ecosystem is very limited [1]. The knowledge of microbiota has increased over the last years with the emergence of next-generation sequencing technology and bioinformatics [2].

The development of many diseases can be triggered by the microbiota composition [3–4], This knowledge has been mainly generated in human research although other species are also being investigated [5–6] not only as the species of interest but also as a model to mimic the role of human microbiota in relation to disease. Thus, the pig digestive microbiota is a topic of research due to its relevance as a main organ suffering many swine diseases and its role can be critical to understand the natural barriers against foreign invaders. Finally, the porcine microbiota can be affected by many factors such as stress, diet, management practices and antimicrobial compounds that could be key factors in the pathogenesis of many digestive disorders [7–8].

The use of antimicrobials in veterinary medicine could be associated with the emergence of bacteria resistant to antimicrobials in food producing animals [9]. Fortunately, the use of antimicrobials in veterinary medicine is decreasing significantly across Europe due to the application of new programs not only at national but also at European level, aiming to reduce the occurrence of multi-resistant pathogenic bacterial strains [10–11]. Antimicrobials are usually prescribed for therapy and metaphylaxis in pig medicine, which implies treatment of many animals with different clinical conditions [12]. In the case of piglets during the post-weaning period, it is highly probable to observe clinical outbreaks of digestive disorders where *Escherichia coli* is one the main pathogens involved. Up to date, in many European countries, the use of antimicrobials either in feed or in water has been an essential tool applied in preventive medicine programs to safeguard animal health during the nursery period [13]; nevertheless, this use is in disagreement with the European legislation about prudent use of antimicrobials in livestock. Thus, it is necessary to develop new alternatives not based on the use of antimicrobials to control disease such as gut health promoting feed additives to help avoiding digestive disorders during the nursery period. In this study, we investigate the effect of a feed additive that contains coated short chain fatty acids (butyrate and propionate), medium chain fatty acids (caprylic, capric and lauric acid) and essential oil components (thymol, cinnamaldehyde and eucalyptus oil). These components have been linked to improve gut health [14–20] but its effect on microbiota is not yet well characterized. Thus, the main goal of this research work is to decipher the effect of a feed additive on swine bacteria microbiota and compare it with pigs treated and untreated with antimicrobials.

## Material and methods

### Animals and sampling

All procedures involving animals followed EU normative (Directive 2010/63/EU). A total of 30 post-weaning piglets were selected from a production farm with recurrent problems of post-weaning colibacilosis (Masia Borras farm, Bellvis d´Urgell, Lleida, Spain). These animals were allocated in an experimental farm (CEP, Torrelameu, Lleida, Spain). All the animals received a diet with Zn oxide (3000 ppm) during the first week after weaning. After this first week, they were split in three experimental groups of 10 piglets each that received different diets. Thus, the control group received a standard post-weaning diet (Table 1) without antibiotics or additives, whereas the antibiotic group received the same diet as the control group but with amoxicillin (15 mg/kg bw/day) and colistin sulphate (5 mg/kg bw/day). Finally, the feed additive group received the same diet as the control group but with a feed additive (Sanacore-EN, Nutriad International N.V.) at a dose of 3 Kg/tonne that contains 71% of coated short chain fatty acids (butyrate and propionate), 10% of medium chain fatty acids (caprylic, capric and lauric acid) and 19% of essential oil components (thymol, cinnamaldehyde and eucalyptus oil).

**Table 1:** Calculated composition of the diet used for piglets.

Diet	
DM, g/Kg	884.2
DM basis, g/Kg	
Protein	182
Fat	53.1
Crude fiber	31.5
Ash	49.9
Nitrogen free extract	467.1
ME, MJ/kg	13.92

Each experimental group was allocated in one pen. Daily feed consumption was registered for each pen. Faeces were taken at 0, 15 and 30 days after beginning the trial and they were immediately frozen at -80°C. Animals were weighed at days 0, 15 and 30 of the trial and average daily weight gain (ADWG) and feed conversion rate (FCR) were calculated for each animal and pen (experimental group), respectively. Briefly, ADWG was calculated as the weight at the last studied time point (30 days of the trial) minus the weight at first selected time point (0 days of the trial) divided by the days lapsed between both time points. FCR was calculated as the feed consumption at pen level during the trial divided by the increase of weight observed for the animals included in each group. At the end of the experiment, piglets were euthanized with an intravenous overdose of penthobarbital and samples of duodenum, jejunum, ileum and cecum were immediately fixed in 10% formaldehyde for histopathological analysis.

The animal clinical status was registered daily. The presence of diarrhoea was specially monitorized using the following clinical score: 0, 1, 2 and 3 for normal faeces, softy faeces (diarrhoea is not clear but faeces could be more consistent), low consistency of faeces and watery faeces, respectively. All studies were approved by the ethical committee of Universitat de Lleida and the Departament d’Agricultura, Ramaderia, Pesca, Alimentació I Medi rural (Section of Biodiversity and hunting) under licence DAAM 7700.

### Morphometric analysis

Tissue samples for the morphometric study were dehydrated and embedded in paraffin, sectioned at 4 μm, and stained with hematoxylin and eosin. Morphometric measurements were performed with a light microscope (BHS, Olympus, Barcelona, Spain). Measurements were taken in 10 well-oriented villi and crypts from each intestinal section of each animal. The villus height and crypt depth were measured using a linear ocular micrometer (Olympus, Microplanet). Villus:crypt ratio was calculated by dividing villus height by crypt depth. All morphometric analysis was carried out by the same pathologist, who was blinded to the treatments.

### DNA extraction, PCR amplification and massive sequencing

Five pigs for each of the groups (control, antibiotic and feed additive) were selected for the microbiota analysis. These animals were clinically healthy (without taking into account the diarrhoea score) and had an average productive performance inside their experimental group (data from all the animals are provided as supplementary material (S1 Table)). Bacterial DNA was extracted from 0.2 g of faeces using the Power Faecal™ DNA isolation kit (MO BIO) under manufacturer’s conditions The quality and quantity of DNA was evaluated on a Nanodrop. DNA samples (100 μl) were stored at -20°C until further processing.

V1-V2 regions of 16S rRNA gene were amplified with barcoded forward primer F27 and reverse primer R338, with sequencing adaptors at the 5′ end. Briefly, each of the primers contained a unique barcode, so that the derived sequences can be sorted into the respective sample bioinformatically in downstream analysis. PCR mixture (25uL) contained 5 μl of DNA template (~5 ng), 5 μl of 5x Phusion® High Fidelity Buffer, 2.5 μL of dNTPs (2 nm), 0.2 μM of each primer and 0.5 U of Phusion® Hot Start II Taq Polymerase (Thermo Fisher). The PCR thermal profile consisted of an initial denaturation of 30 sec at 98°C, followed by 30 cycles of 15 sec at 98°C, 15 sec at 55°C, 20 sec at 72°C and a final step of 7 min at 72°C. To assess possible reagent contamination, each PCR reaction included a no template control (NTC) sample. The PCR product was purified and concentration and quality were determined for each amplicon using Qubit™ fluorometer and Agilent Bioanalyzer 2100. Barcoded amplicons were sequenced on an Ion Torrent Personal Genome Machine (PGM) with the Ion 318 Chip Kit v2 (Life Technologies) and the Ion PGM™ Sequencing 400 Kit (Life Technologies) under manufacturer’s conditions.

### Quality control, OTU assignment, composition, diversity and functional analyses

Raw sequencing reads were demultiplexed, quality-filtered and analyzed using QIIME 1.9.1 [21]. Reads included had: a length greater than 300 bp; a mean quality score above 25 in sliding window of 50 nucleotides; no mismatches on the primer; and default values for other quality parameters. Quality-filtered reads were processed using *vsearch* v1.1 pipeline [22]: a first de-replication step was applied, followed by clustering into operational taxonomic units (OTUs) at 97% similarity with a *de novo* approach. Finally, chimera checking was performed using uchime *de novo*. Raw OTU table was transferred into QIIME 1.9.1 and taxonomic assignment of representative OTUs was performed using the RDP Classifier or equivalent against Greengenes v13.8 database [23]. Alignment of sequences was performed using PyNast as default in QIIME pipeline, with an extra filtering step in aligned and taxonomy-assigned OTU table to filter-out sequences that represent less than 0.005% of total OTUs. Downstream analyses were performed at the same depth per sample to standardize for unequal sequencing depth of the samples.

Samples were grouped according to treatment (control, antimicrobial and feed additive) initially, and further analyzed based on day of the trial. Analysis was performed at different taxonomical levels separately (phylum, family and genus). Diversity indices were calculated on rarefied 16S rRNA gene sequence data for all samples at 97% similarity using QIIME (*alpha_diversity*.*py* script). Thus, two different metrics have been used for alpha diversity: observed species (observed OTUs), and Shannon index. Statistical significance was assessed with 999 permutations using the non-parametric Monte Carlo permutation test and *compare_alpha_diversity*.*py* QIIME script. The p-value was corrected through false discovery rate. *P* < 0.05 was considered statistically significant.

To compare microbiota composition among samples (qualitative and quantitative), a beta diversity analysis was performed considering the presence/absence of OTUs in each sample (Unweighted UniFrac) and the presence/absence and the abundance of each detected OTU (Weighted UniFrac). Weighted and unweighted UniFrac phylogenetic distances were used to generate the beta diversity distance matrices and calculate the degree of differentiation among the samples. Samples were grouped according to different characteristics to test them as possible factors leading to clustering.

Principal coordinate analysis was carried out on each group and resampling was performed repeatedly on a subset of the available data of each sample evenly (jacknifing) to measure the robustness of individual clusters in PCoA plots. Bray-Curtis and both weighted and unweighted UniFrac distance metrics were used to create PCoA plots and to generate Unweighted Pair Group Method with Arithmetic Mean (UPGMA) trees (*jackknifed_beta_divesity*.*py* QIIME workflow script) [24–25], ANOSIM and ADONIS statistical methods from QIIME 1.9.1 were applied to evaluate if some variables were clustered and to which extent. Finally, a linear discriminant analysis (LDA) effect size (LEfSe) [26] was carried out to identify taxa whose abundance is differentially abundant between experimental groups (α = 0.05 and with an LDA score > 3.0). The datasets analyzed during the current study are available in the SRA NCBI repository under the Bioproject accession number PRJNA445806.

### Statistical analyses

All of the statistical analyses, with the exception of microbiota analysis, were performed using SPSS software, version 15.0 (SPSS Inc., Chicago, Illinois, USA). The alpha level used for determination of significance for all analyses was *P* < 0.05, with statistical tendencies reported when *P* < 0.10. The individual pig was used as the experimental unit. The variables included in the statistical analyses were classified as categorical (experimental group), ordinal (diarrhea level) or continuous (average daily gain and morphometric data in the histopathological analysis). Shapiro Wilk´s and Levene tests were used to evaluate the normality of the distribution of the continuous variables and the homogeneity of variances, respectively. Contingency tables (Chi-square or Fischer exact tests) were used to test the association between nominal and ordinal variables. To study the association between nominal variables with the continuous non-normally distributed variables (morphometric data), the Wilcoxon test (with the U Mann-Whitney test to compare each pair of values) was used. To analyse the association between continuous normally distributed variables (average daily gain) and nominal variables, an ANOVA test (with Bonferroni test to compare each pair of values) was used.

## Results

### Clinical evolution and productive performance

**Fig 1:**
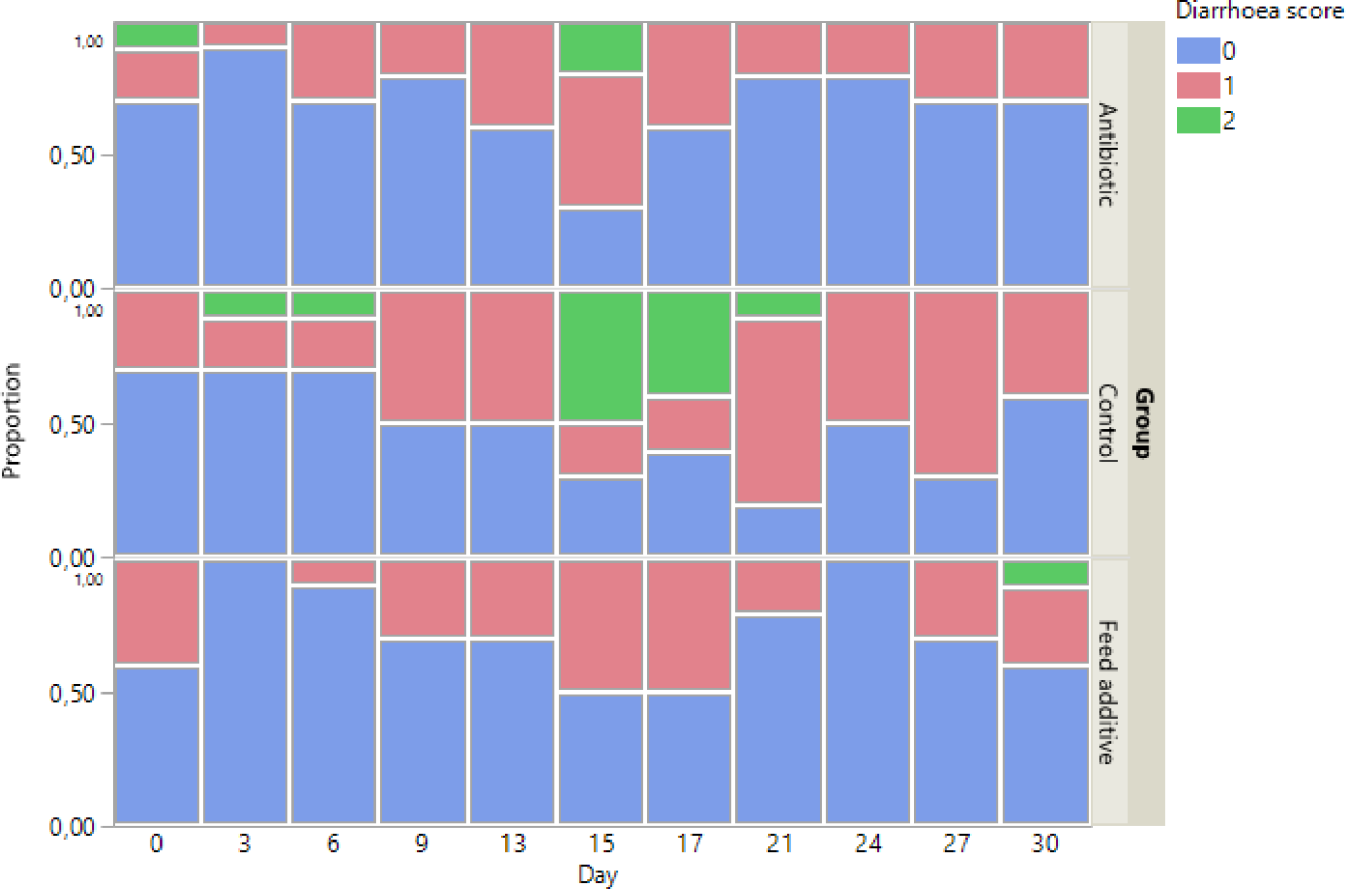
Evolution of diarrhoea score throughout the trial for control, antibiotic and feed additive group. The proportion of animals (from 0 to 1) in each experimental group by day is represented depending on the diarrhoea score. Thus, 0, 1, 2 and 3 for normal faeces, softy faeces (diarrhoea is not clear but faeces could be more consistent), low consistency of faeces and watery faeces, respectively.

No digestive outbreaks were observed throughout the trial (score = 3). However, between the second and third week, some animals, the majority belonging to the control group presented diarrhoea (score = 2) (Fig 1). A total of four animals, three from the feed additive group and one from the control group were excluded from the study and they were not used to carry out any analysis (data from all the animals are provided as supplementary material (S1 Table). In particular, two animals of the feed additive group presented loss of corporal condition (apparent loss of weight) without showing clinical signs (eg: fever). Additionally, one animal from the control and other from the feed additive group exhibited fever and they received antimicrobial treatment and were allocated in nursery pens.

The control group showed the highest diarrhoea score (Fig 1) throughout the trial showing statistical significant differences compared with the antibiotic and feed additive groups only at 15 and 17 days post-beginning the trial (p<0.05). The feed additive group showed the lowest level of diarrhoea score during the trial and the values were quite similar to the antibiotic group across the trial without showing statistical significant differences between them (p>0.05).

Differences in average feed daily intake (AFDI) were observed between groups. The calculated values for the control, antibiotic and feed additive groups were 739, 677 and 607 gram/day, respectively. On the other hand, differences in the average daily weight gain (ADWG) were also observed between groups. The ADWG was 0.53±0.05, 0.48±0.06 and 0.44±0.05 Kg/day for the control, feed additive and antibiotic group, respectively. The observed differences in ADWG were only statistically significant between the control and antibiotic group. Moreover, the feed conversion rate was 1.40, 1.44 and 1.52 for the feed additive, control and antibiotic groups, respectively. Finally, animals with an ADWG close to the average value for each experimental group were selected as the most suitable ones for inclusion in the microbiota analysis as detailed before.

### Morphometric results

**Fig 2:**
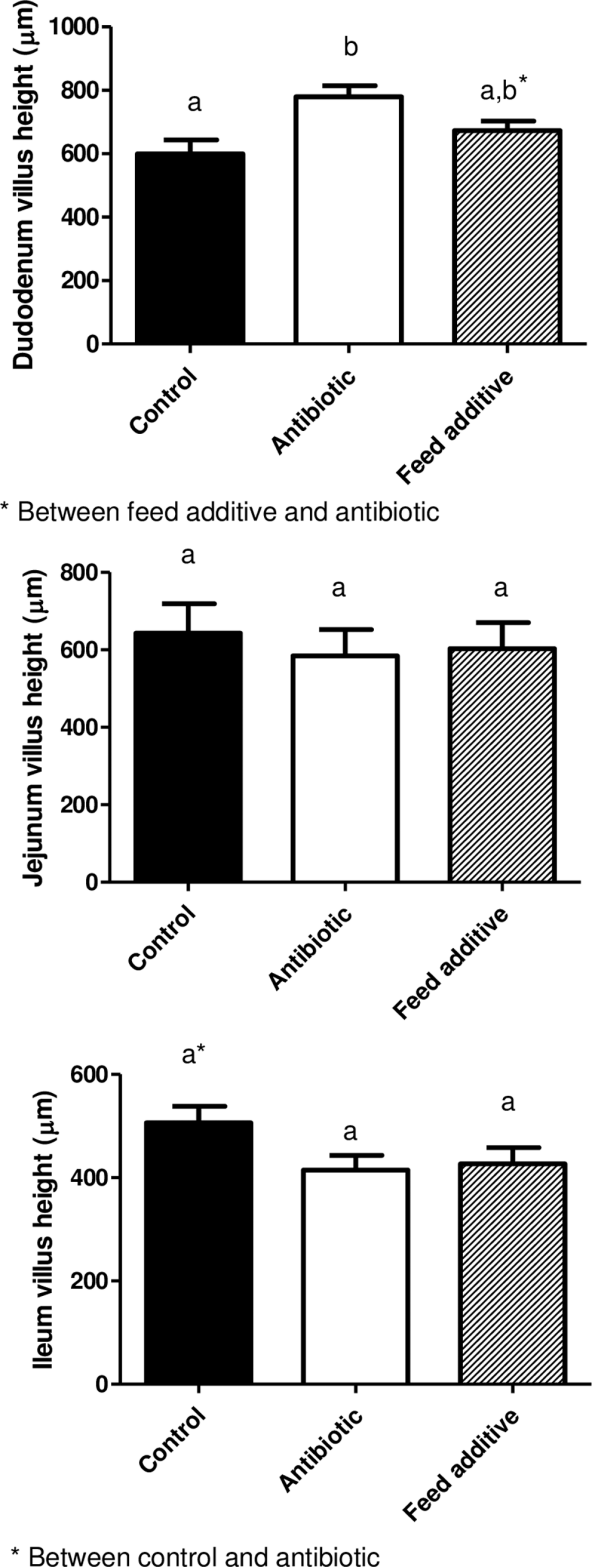
Villus height observed for duodenum, jejunum and ileum in the control, antibiotic and feed additive groups (medium and SEM). Different letters means statistical significant differences (p<0.05). * mean statistical tendency.

**Fig 3:**
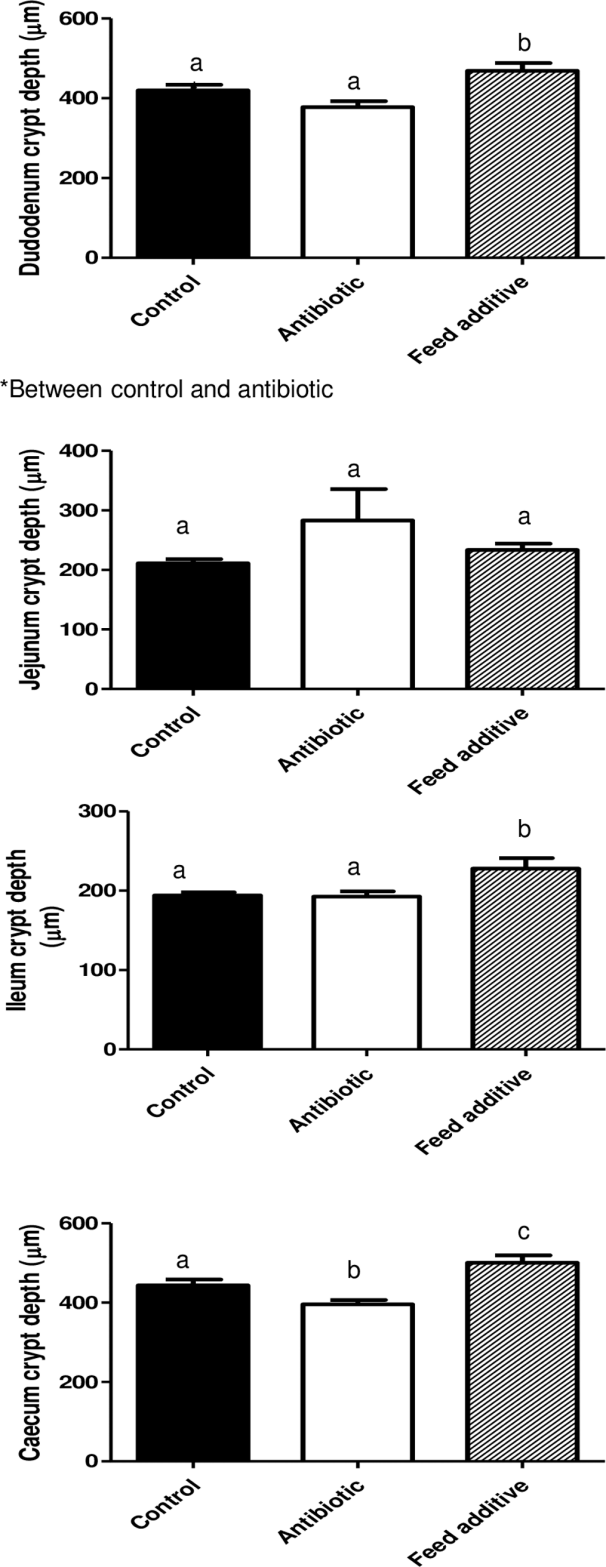
Crypt depth observed for duodenum, jejunum, ileum and caecum in the control, antibiotic and feed additive groups (medium and SEM). Different letters means statistical significant differences (p<0.05). * mean statistical tendency.

**Fig 4:**
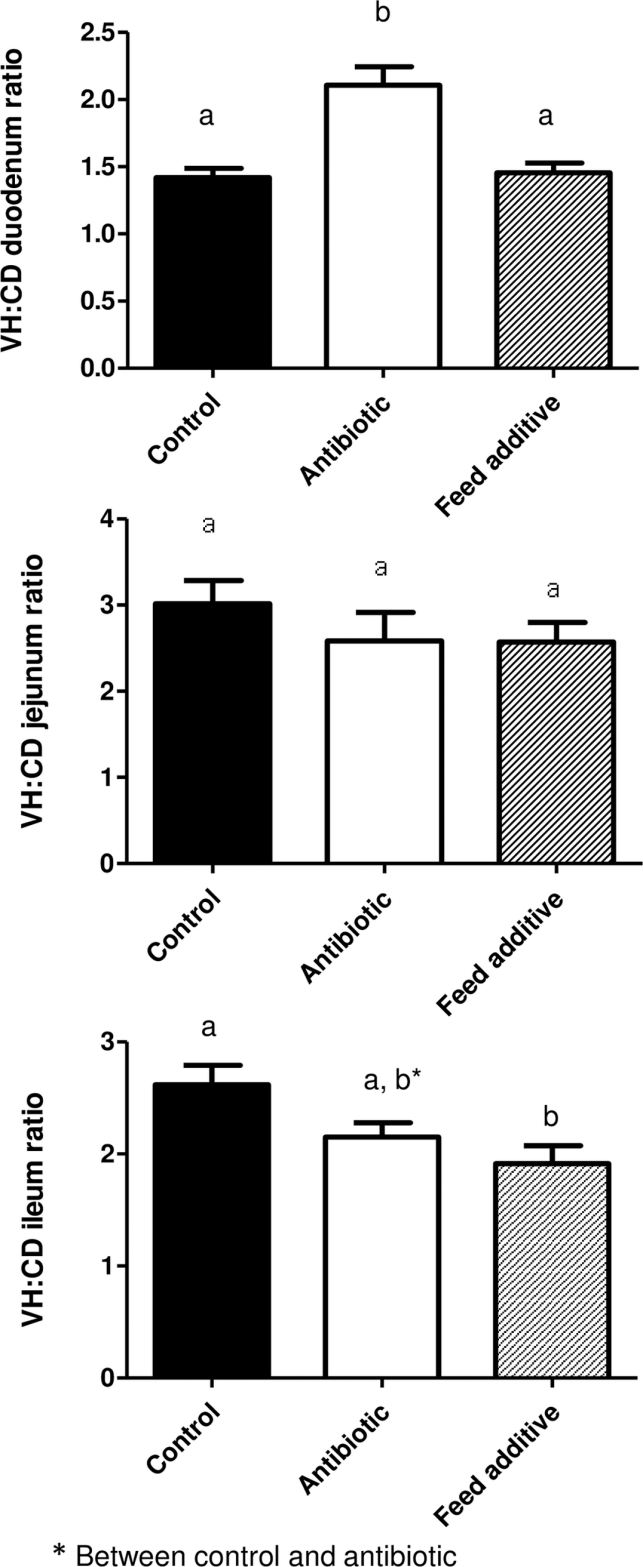
Villus versus crypt height ratio observed for duodenum, jejunum and ileum in the control, antibiotic and feed additive groups (medium and SEM). Different letters means statistical significant differences (p<0.05). * mean statistical tendency.

Morphometric determinations are detailed in Figs 2–4. Thus, the villus height (VH) was not significantly different (p>0.05) between the three experimental groups in all the intestinal segments (Fig 2), with the exception of the duodenum where the VH was larger in the antibiotic group compared with the control (p<0.05) and feed additive groups (statistical tendency). On the other hand, the crypt depth was significantly bigger (p<0.05) in the feed additive than in the control and antibiotic groups for duodenum, ileum and caecum. For this parameter, no significant differences were observed between the control and antibiotic group except for the caecum (Fig 3). Finally, the villus height:crypt depth ratio (VH:CD ratio) was smaller in the feed additive than in the control (p<0.05) and antibiotic group (statistical tendency) for ileum. Conversely, this parameter was bigger (p<0.05) in the antibiotic than in the control and feed additive groups in the case of duodenum (Fig 4).

### Microbiota results

After quality control and extra-filtering filter steps, our samples ranged from 22,249 to 50,418 sequences per sample, with a median of 35,872 sequences per sample. Downstream analyses were performed at a sequencing depth of 22,000 sequences per sample.

#### Age-dependent evolution of digestive microbiota- Evolution in the control group

**Fig 5:**
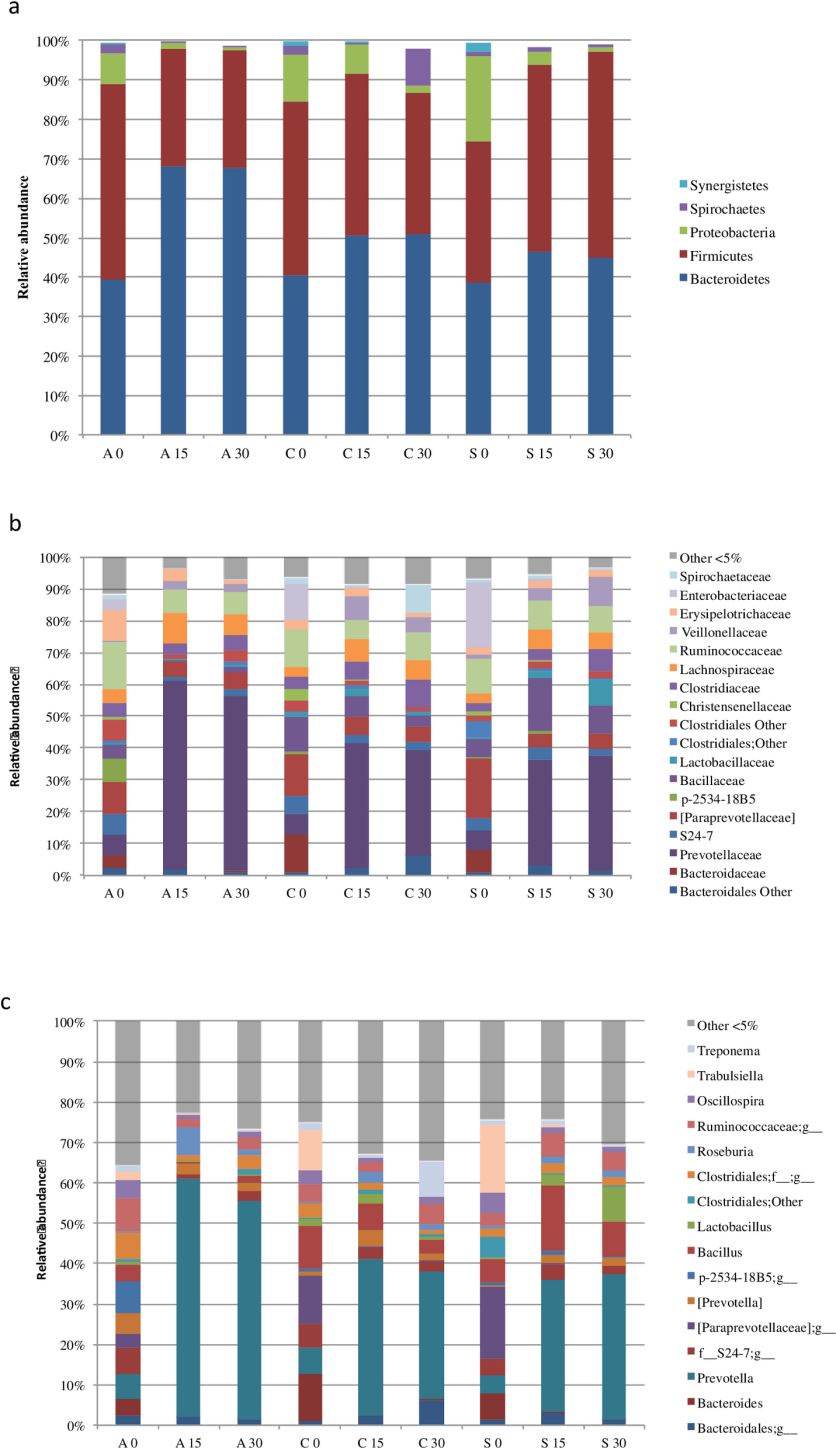
Digestive microbiota from antibiotic (A), control (C) and feed additive (S) treated piglets. The mean relative abundance (%) of OTUs found at phylum (a), family (b) and genus (c) level in faecal samples is presented. X Y means experimental (control-C, feed additive- S, antibiotic-A,) and day group (0, 15 and 30), respectively.

OTUs were identified in all samples by clustering sequences at 97% sequence similarity. The maximum number of OTUs clustered per pig was 854 OTUs (Table 2). At the taxonomy level, we found 15 phyla, 24 classes, 37 orders, 66 families and 126 genera of bacteria. The relative abundance of the OTUs found in the digestive microbiota by age in the different experimental groups is represented in Fig 5. *Proteobacteria* represented a mean of 13.8%, *Bacteroidetes* 39.6% and *Firmicutes* 43.11% in the phyla found in digestive microbiota of the youngest pigs, (Fig 5A). *Spirochaetes* (1.7%) and *Synergestetes* (1.2%) are two phyla with more than 1% of relative abundance. The *Enterobacteriaceae* family was the most abundant within *Proteobacteria*, representing 11.6% of all the families found (91% of the assigned as *Proteobacteria* phylum). The most abundant family found was *Paraprevotellaceae* with 13.7%, which represented 35% of the sequences in the *Bacteroidetes* phylum. On the other hand, *Ruminicoccaceae* was one of the most abundant family (12.4%) that represented 29% of the *Firmicutes* phylum. Moreover, this phylum was more homogeneously divided in different families, and being equally represented *Bacillaceae* (6.9%), *Erysipelotrichaceae* (4.8%), *Lachnospiraceae* (3.5%) and *Clostridiaceae* (3.4%) (Fig 5B).

**Table 2:** Alpha diversity indexes obtained for the individual samples and mean values for all the samples and samples grouped by time point or treatment. C, S and A are control, feed additive and antibiotic group, respectively.

Alpha Diversity index	Group	Mean value by group and time	Standard deviation	Global mean	Time	Mean by time	Group	Mean by group
Observed Species								
	C_0	454.5	120.6	672.6	t_0	487	C	673
	S_0	421.7	153					
	A_0	586.2	122.8					
	C_15	709.9	38.9		t_15	729	S	702
	S_15	843.1	99.7					
	A_15	633.9	111.8					
	C_30	853.8	96.4		t_30	801	A	643
	S_30	842.3	127.7					
	A_30	708.3	46.4					
Shannon Index								
	C_0	5.5	0.9	6.61	t_0	5.8	C	6.6
	S_0	5.5	1					
	A_0	6.4	0.5					
	C_15	6.9	0.4		t_15	6.8	S	6.5
	S_15	6.9	0.7					
	A_15	6.6	0.2					
	C_30	7.3	0.4		t_30	7.1	A	6.7
	S_30	7.1	0.4					
	A_30	7	0.2					

In general terms, age modifies significantly the microbiota of the piglets. Thus, within the phyla found in digestive microbiota of pigs from the first week post-weaning (day 0 of this trial) until 37 days post-weaning, *Proteobacteria* decreased steeply from 13.8% to 1.7%. However, *Firmicutes* remained constant (close to 40%) and *Bacteroidetes* increased from 40% to 51% from day 15 onwards. Interestingly, the largest increased was observed for *Spirochaetes* during this period of time (from 1.7% to 9.4%). Within *Bacteroidetes*, the *Prevotellaceae* family increased from 6.5% to more than 33% from day 15 onwards and the *Bacteroidaceae* and *Paraprevotalleceae* family decreased from 6.6% to almost 0% and 13% to 4.9%, respectively during the same period of time (Fig 5).

#### Effect of the different treatments on the digestive microbiota: Focus on alpha diversity

To unravel the differences in digestive microbiota in piglets receiving different treatments (control, antibiotic and feed additive), we analyzed the relative abundance of OTUs at three main levels (phylum, family and genus) by grouping also samples according to the treatments. Fig 5 shows the average relative abundance per experimental groups at phylum (Fig 5A), family (Fig 5B) and genus level (Fig 5C). Thus, there is a relative increase in *Bacteroidetes* in conjunction to a decrease in *Firmicutes* in animals receiving antibiotic treatment from day 15 onwards. The increase in *Bacteroidetes* in antibiotic-treated pigs corresponds to a higher abundance of the family *Prevotellaceae* (Fig 5A).

**Fig 6:**
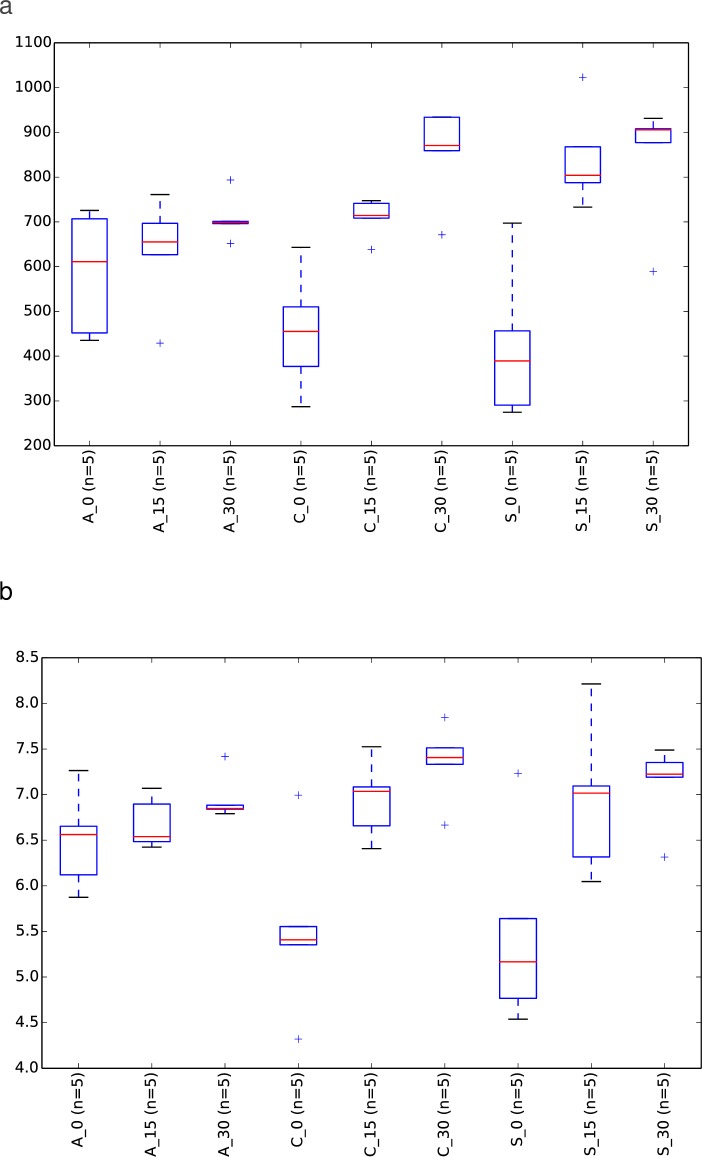
Alpha diversity on samples analyzed by experimental group and time point. Alpha diversity was compared between groups by measuring different metrics: Observed species (a) and Shannon Index (b). X_Y means experimental (control-C, feed additive- S, antibiotic-A,) and day group (0, 15 and 30), respectively.

**Fig 7:**
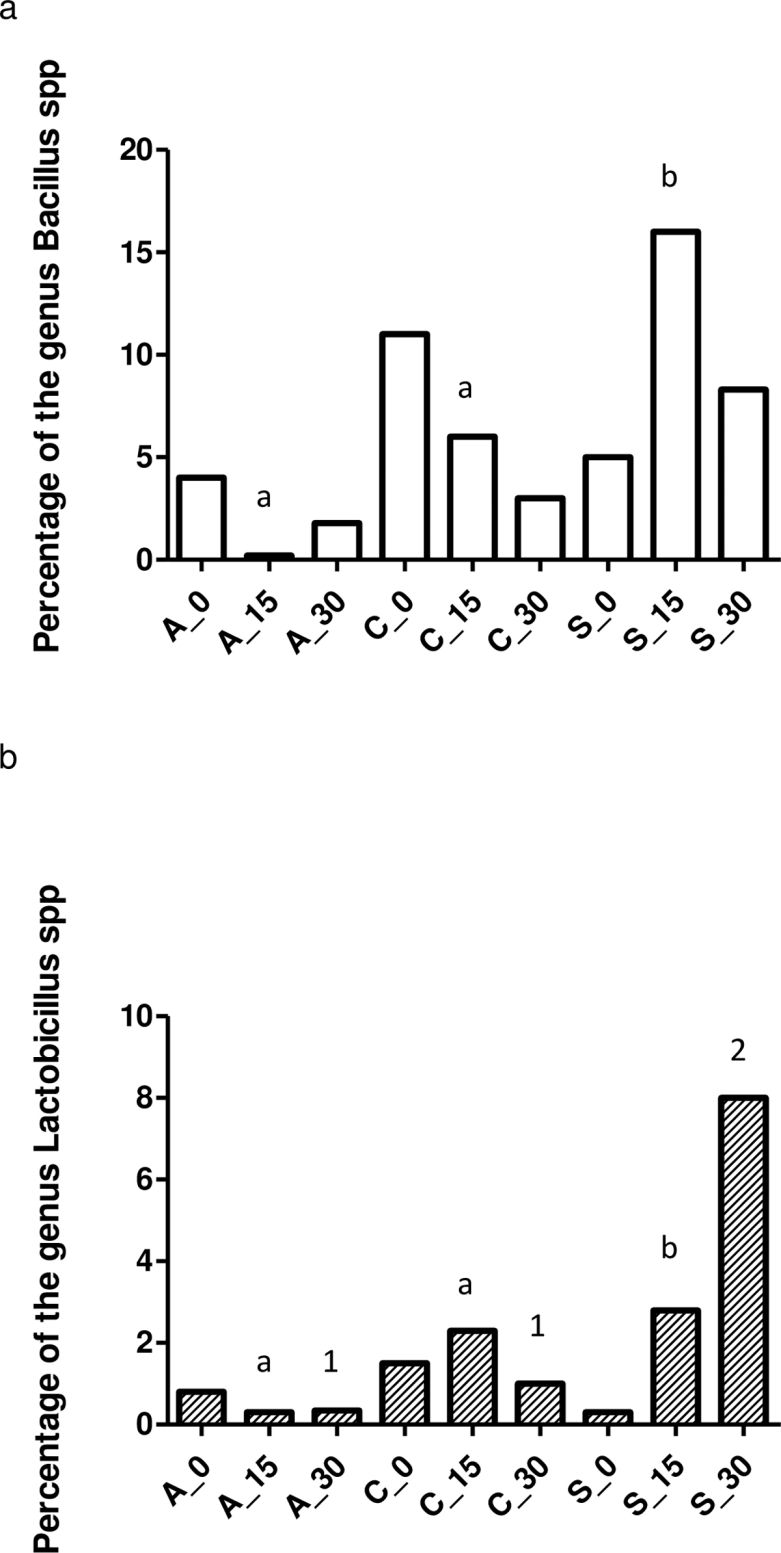
Percentage of the genus *Bacillus* (a) and *Lactobacillus* (b) spp grouped based on the treatment applied at day 0, 15 and 30 post-beginning the trial. X_Y means experimental (control-C, feed additive- S, antibiotic-A,) and day group (0, 15 and 30), respectively. The statistical differences are shown between groups inside each sampling time. Different letters and numbers means statistical significant differences for 15 and 30 days of the trial, respectively (p<0.05).

The oldest the animal, the highest bacterial diversity (both, observed species and Shannon Index) observed for the control and the feed additive groups (Table 2 and Fig 6). However, this diversity was very similar in the antibiotic group throughout the trial, which is clearly represented by the boxplots from the median values in Fig 6. Finally, a large difference was observed in the evolution of bacteria of the genus *Bacillus* and *Lactobacillus spp* between the experimental groups throughout the trial. Thus, a clear increase in abundance of both bacterial genera was detected within the feed additive group versus the antibiotic and control groups (Fig 7). This difference is even more remarkable for the genus *Lactobacillus spp* at the last time point of the trial.

Herein, the basal homogeneity in microbiota is guaranteed, since not significant differences were detected between the three groups at the beginning of the trial (day 0). These results allowed further comparison at the different time points. It was observed significant (p<0.05) differences in alpha diversity between sampling times (0, 15 and 30) and experimental groups (control, antibiotic and feed additive) in this trial. Finally, it is only observed a statistical tendency (p = 0.07) in alpha diversity between the group feed additive and antibiotic at day 15 probably due to the low statistical potency (5 pigs) available when it is compared the time and group at the same time. Finally, a significant increase (p<0.05) was observed in the abundance of *Lactobacillus spp* in the feed additive group versus the control and antibiotic one from day 15 onwards whereas it was only observed a significant increase of the *Bacillus spp* in the feed additive group versus the control and antibiotic one at day 15 of the trial (Fig 7).

#### Effect of the different treatments on the digestive microbiota: Focus on beta diversity

**Fig 8:**
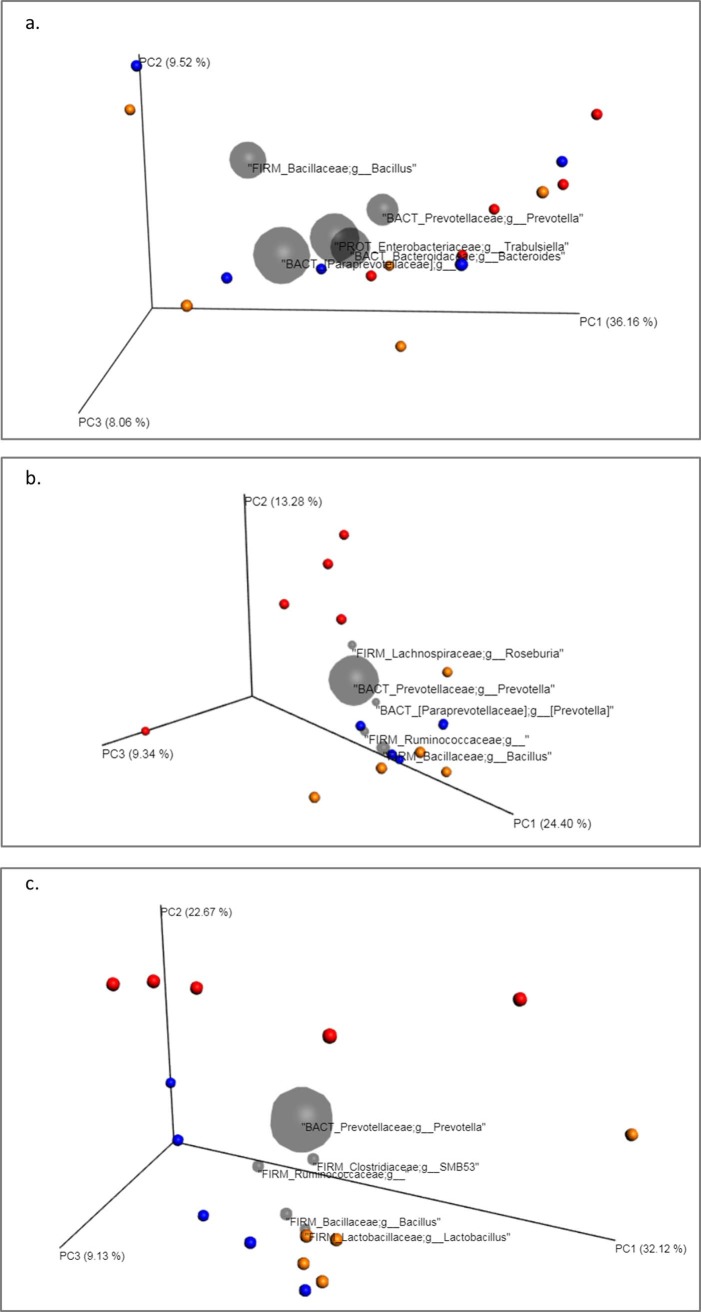
Principal Component Plots (jackknifed) representing beta diversity on samples. Beta diversity of faecal samples of piglets was computed through unweighted UniFrac analysis for control (blue), antibiotic (red) and feed additive treated (orange) piglets at day 0 (a), 15 (b) and 30 (c) of the trial.

**Fig 9:**
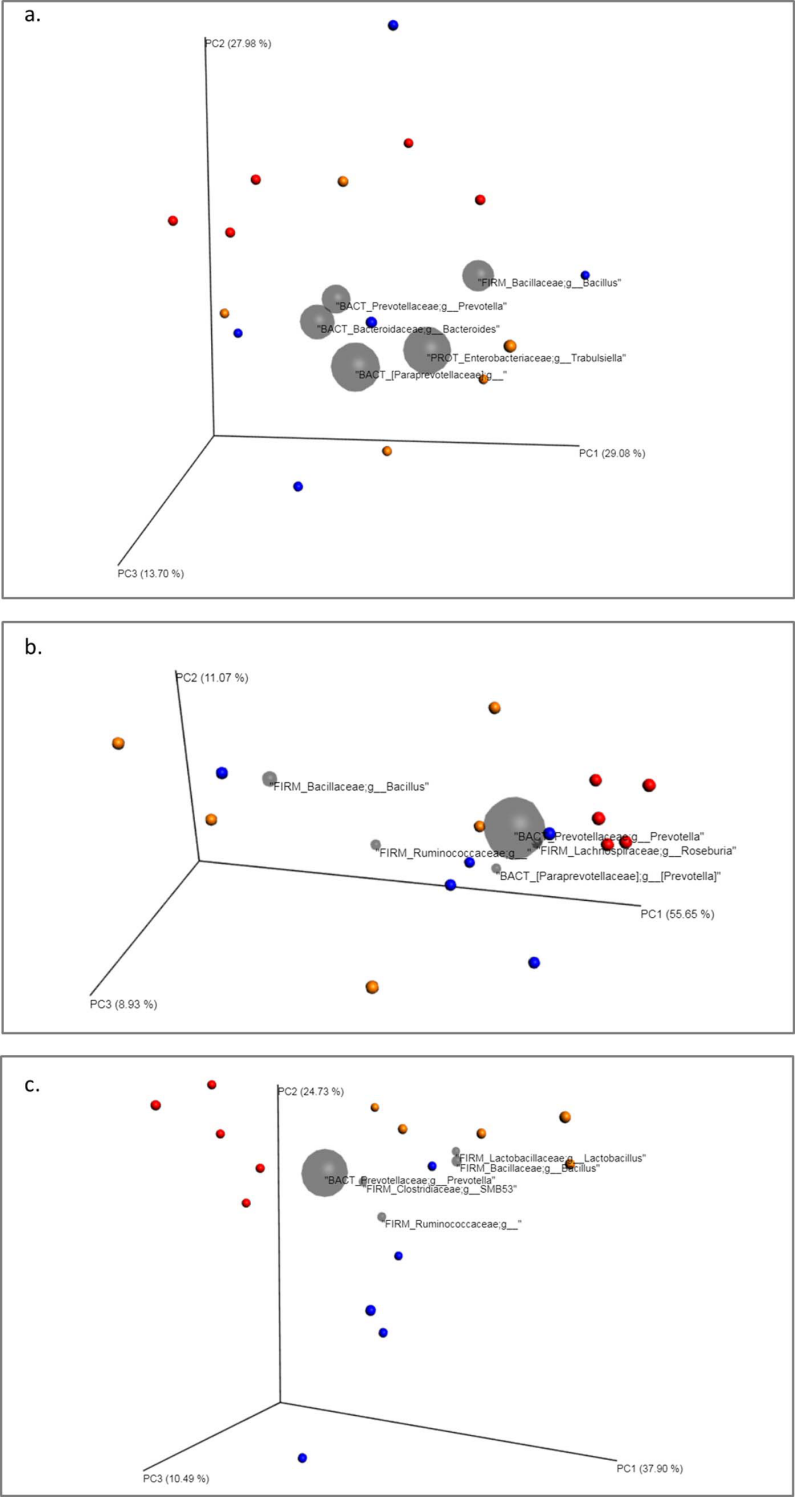
Principal Component Plots (jackknifed) representing beta diversity on samples. Beta diversity of faecal samples of piglets was computed through weighted UniFrac analysis for control (blue), antibiotic (red) and feed additive treated (orange) piglets at day 0 (a), 15 (b) and 30 (c) of the trial.

The PCoA plots obtained are depicted in Figs 8 and 9. Regarding the treatment applied, distances among groups were calculated and results demonstrated statistical differences at day 15 and 30 of the trial in both, the weighted and the unweighted UniFrac analysis (Table 3). At day 15, grouping by treatment significantly explained 26% of the variation in UnWeighted UniFrac plot and 35% in Weighted UniFrac. At day 30, these values were 35% and 46% respectively, showing both different composition and community structure of the microbiota according to the treatment applied. On the other hand, the ANOSIM R-value ranges from -1 to 1. A value close to 0 indicates that there are no differences between populations, while a value close to 1 indicates that there are differences between the groups compared. The ADONIS test was significant for days 15 and 30 and for both Weighted and Unweighted Unifrac distance matrices.

**Table 3:** Results for ANOSIM and ADONIS tests in the comparison of the microbiota composition for time-point and treatment.

ANOSIM test			UnWeighted_0	UnWeighted_15	UnWeighted_30	
Sample size	15	Test result	0.001	0.510	0.454	
Number of groups	3	*P*-value	0.395	0.002^a^	0.002^a^	
			Weighted_0	Weighted_15	Weighted_30	
Sample size	15	Test result	0.002	0.312	0.660	
Number of groups	3	*P*-value	0.438	0.016^a^	0.001^a^	
ADONIS test	Df	Sums of square	Means square	F model	R^2^	*P*-value
UnWeighted_0	2	0.27694	0.13847	1.1599	0.16199	0.255
Residuals	12	1.43264	0.11939		0.83801	
Total	14	1.70958			1	
UnWeighted_15	2	0.35211	0.176053	2.1702	0.26563	0.001^a^
Residuals	12	0.97346	0.081122	0.73437		
Total	14	1.32556	1			
UnWeighted_30	2	0.41869	0.209346	3.2941	0.35443	0.001^a^
Residuals	12	0.76261	0.063551	0.64557		
Total	14	1.1813	1			
Weighted_0	2	0.11667	0.058337	0.97899	0.14028	0.454
Residuals	12	0.71507	0.059589	0.85972		
Total	14	0.83175	1			
Weighted_15	2	0.16583	0.082917	3.1706	0.34573	0.014^a^
Residuals	12	0.31383	0.026152	0.65427		
Total	14	0.47966	1			
Weighted_30	2	0.20104	0.100522	5.133	0.46106	0.001^a^
Residuals	12	0.235	0.019583	0.53894		
Total	14	0.43604	1			

^a^ means statistically significant differences (p<0.05)

## Discussion

During the last decades, the use of antimicrobials has been compromised due to the emergence of bacteria resistant to a wide range of antibiotics. Several studies have demonstrated shifts in the gut microbiota of pigs after supplementing the diet with antimicrobials [7,8,27]. Moreover, an increase in the abundance and diversity of resistance genes has been described under a particular medication, even for antimicrobial families not administered to the animals [28]. Thus, in recent years, there is a growing interest in the development of products as alternatives to antibiotics. These alternatives must combine a positive effect in the gut microbiota with an improvement in immunity, health status and growth performance of the animals. Probiotics have been suggested as good candidates for seeking this effect. However, these probiotics are frequently microorganisms that have to reach the site of action at the correct concentration, compete with the natural microbiota, and must colonize the gut to fulfill a longtime effect in the animal. Additionally, some bacterial species use as probiotics may be prone to acquire resistance genes due to natural processes of horizontal gene transfer, such as transformation, conjugation or transduction [29]. From this point of view, active ingredients or metabolites that can modulate the microbiota, and potentiate the immune system appear to be a safer alternative not only to antibiotics but also to probiotics.

The stressors associated with weaning and the concomitant reduction in feed intake in early life can result in the atrophy of villi, leading to the reduction of the surface area for nutrient absorption and compromised gut barrier function ultimately leading to causing diarrhoea [30]. Moreover, weaning is associated with intestinal inflammation and a systemic proinflammatory response [31–32]. For these reasons, weaning is a risk factor associated with increasing incidence of digestive disorders, where bacterial diseases usually play a major role. In the past, the use of antimicrobials has been an essential tool for preventing and controlling digestive disorders during weaning. However, as previously mentioned, alternatives to the prophylactic use of antimicrobials are urgently needed. Results from this study have shown that post-weaned pigs fed a diet supplemented with one of these alternative products exhibited significantly lower incidence of diarrhea and a better feed conversion rate than the control group. It is evident that these results must be interpreted with caution herein, since there are no replicates in our experimental design. In any case, the feed conversion rate response to this alternative was not probably significant in this study due to the low number of replicates. Hence the improvement observed for this parameter is consistent with that observed in larger studies [33] with greater number of pigs that were designed to evaluate the performance response and establishes that pigs in this study were responding to this alternative in an expected way. On the other hand, two animal of the additive group were excluded due to loss of corporal condition without reaching an exact diagnosis. However, a significant increase of runt piglets has not been observed in animals consuming this feed additive versus the control ones in an experiment using large number of animals [33]. Thus, we believe that our finding is an event neither related with the feed additive consumption nor with any other relevant disease that can affect the results obtained in this research work.

Interestingly, the results reported here also indicate that this additive can modify the morphology of the small and large intestine, since the crypt depth was significantly bigger in the feed additive than in the control and antibiotic groups for duodenum, ileum and caecum. It is very tempting to link these differences in the gut morphology between groups with the improvement observed in productive parameters; however we were unable to find bibliographic references that could directly support this affirmation. In any case, it is clear that viruses that affect crypts in the gut have a major negative impact in gut physiology and on productive performance than those viruses that only infect cells located in the villus. Thus, to assure crypt integrity is a hallmark to improve recovery of affected animals for gut pathogens [34]. Finally, the effect on gut morphology cannot be analyzed separately from other concomitant effects, such as the observed for the microbiota. In fact, the improvement observed in health status and productive performance is probably associated to a plethora of effects at gut level.

In the control group, we observed a significant enrichment in *Prevotellaceae* from day 0 to day 15 in contrast with the decrease in *Bacteroidaceae* which was further maintained for both families from day 15 to day 30. Since day 0 of our experimental design was set at one week post-weaning, the gut microbiota may have undergone a developmental process of adaptation to the new diet during the first few weeks post-weaning. This may also explain the reduction of the *Enterobacteriaceae* family from day 0 to day 15 [35]. Furthermore, whereas *Bacteroides* obtained the energy through fermentation of proteins such as animal fat, *Prevotella* is associated with a plant-rich diet [36] and is a known mucin degrader [37]. On the other hand, it is noteworthy the increase in abundance of the *Spirochaetaceae* family and more specifically the genus *Treponema* observed at day 30 in the control group. Strains of *Treponema* are the causative agent of porcine skin necrosis and ulcers [38]. Several studies have reported the presence of different species of the family *Spirochaetaceae* in animals not treated with antimicrobials [7,39]. Looft *et al*. [8] demonstrated a reduction in this family attributed to the use of cabadox as growth promoter and suggested its inhibitory effect in potential intestinal pathogens, such as *Brachyspira hyodysenteriae*. However, further studies are warranted to decipher the microbial interactions that have triggered this abundance and the real effect in growth performance and intestinal health. Overtime, the gut microbiota matures and also becomes more stable as reported by other studies [40]. On the contrary, a less stable and diverse microbiota may be more predispose to environmental changes, such as diet and, as a consequence, more responsive to prebiotic supplementation [41].

In general, the alpha diversity was higher for the control and the feed additive groups. High microbial diversity has been described to be beneficial to the mucosal surfaces since decreases the opportunity of pathogens colonizing the gut [42]. Several studies have described a reduction in diversity of the faecal microbiota during antimicrobials administration [43] which our results strongly support throughout the trial. Administration of amoxicillin and colistin would have an effect on the *Lactobacillus* spp depletion with a reduction of aerobic and anaerobic bacteria [44], and a reduction of Gram-negative organisms [45], respectively. This correlates with the rapid decrease in abundance of *Proteobacteria* and *Lactobacillus* observed in the antibiotic treated group and the shift in abundance of the *Prevotellaceae* family occupying their niche. Interestingly, Unno *et al*., [39] observed a negative correlation between productive performance and the abundance of the family *Prevotellaceae* species, results which are in agreement with our observation, where the antibiotic group exhibited a worse feed conversion rate than the other two groups.

A dominance of certain *Firmicutes* has been associated to a good gut health [46–47]. In particular, an increment in abundance of the families *Bacillaceae* and *Lactobacillaceae* which correlated with a significant increase in the genus *Bacillus* and *Lactobacillus*, was observed for the animals in the feed additive group, especially at day 30. These genuses are practically depleted in animals treated with antibiotics and found in lower proportion in the control group. In addition, species of *Bacillus* are known to produce different antimicrobial compounds, such as bacteriocins, lantibiotics, polyketides, nonribosomal peptide synthetases, and siderophores [48–49]. Lan *et al*. [50] reported the reduction of the pH in the gut of weaning pigs with a beneficial effect in nutrient digestibility attributed to the presence of lactic acid bacteria (a mixture of *Bacillus*, and *Lactobacillus*). Furthermore, lactic acid bacteria are closely link with strains of the genus *Veillonella* in a natural microbial food-chain [51]. The combination of both has been demonstrated to confer an inhibitory effect to enteropathogenic bacteria by competitive exclusion [52].

## Conclusions

Antimicrobials have been widely used in a prophylactic way to decrease the incidence of digestive disorders during the piglet post-weaning period. Nowadays, there is an urgent need to reduce the consumption of antimicrobials to cope with antimicrobial resistance in livestock. In this research paper, one alternative to antimicrobials, based on a combination of encapsulated short-chain fatty acids, medium-chain fatty acids and protected essential oils, was able to increase bacterial diversity and increase the abundance of *Bacillus* and *Lactobacillus spp* in pig microbiota. This finding helps to understand its mechanism of action in the control of piglet digestive disorders.

## Supporting information

S1 Table Productive performance of the piglets included in the trial. Selected piglets for microbiota analysis are described.
(XLS)
